# Oligo-residual disease in PD-1/PD-L1 inhibitor-treated metastatic non-small cell lung cancer: incidence, pattern of failure, and clinical value of local consolidative therapy

**DOI:** 10.1007/s00262-024-03720-7

**Published:** 2024-06-04

**Authors:** Jinmeng Zhang, Jie Gao, Shanshan Jiang, Jiuang Mao, Li Chu, Xiao Chu, Xi Yang, Yida Li, Tiantian Guo, Yue Zhou, Dayu Xu, Jie hu, Qian Chu, Jianjiao Ni, Zhengfei Zhu

**Affiliations:** 1 Department of Radiation Oncology, Fudan University Shanghai Cancer Center, Shanghai, 200032 China; 2 Department of Oncology, Shanghai Medical College, Fudan University, Shanghai, 200032 China; 3 Shanghai Clinical Research Center for Radiation Oncology, Shanghai, China; 4 Shanghai Key Laboratory of Radiation Oncology, Shanghai, 200032 China; 5 Department of Pulmonary Medicine, Zhongshan Hospital, Fudan University, Shanghai, China; 6 Department of Oncology, Tongji Hospital, Tongji Medical College, Huazhong University of Science and Technology, Wuhan, China

**Keywords:** Non-small cell lung cancer (NSCLC), Radiotherapy, Local consolidative therapy (LCT), Oligo-residual disease (ORD), PD-1/PD-L1 inhibitors

## Abstract

**Objectives:**

To investigate the feasibility and potential clinical value of local consolidative therapy (LCT) in PD-1/PD-L1 inhibitor-treated metastatic non-small cell lung cancer (NSCLC).

**Materials and methods:**

PD-1/PD-L1 inhibitor-treated metastatic NSCLC patients with measurable disease in three academic centers were screened and those with adequate follow-up were included. Oligo-residual disease (ORD) was defined as residual tumors limited to three organs and five lesions evaluated at the best response among patients with partial response or stable disease after PD-1/PD-L1 inhibitors. Oligometastatic and multiple-metastatic disease (OMD/MMD) were similarly classified at baseline. Locoregional interventions, administered after effective treatment of PD-1/PD-L1 inhibitors and before initial disease progression, were defined as LCT. Patterns of initial progressive disease (PD) were classified as involving only residual sites (RP), only new sites (NP), or a combination of both (BP).

**Results:**

Among the 698 patients included, ORD was documented in 73 (47.1%) of 155 patients with baseline OMD and 60 (11.0%) of 543 patients with baseline MMD. With a median follow-up of 31.0 (range, 6.0–53.0) months, 108 patients with ORD developed initial PD, with RP, NP, and BP occurring in 51 (47%), 23 (21.3%), and 34 (31.5%), respectively. Among the 133 patients with ORD, those receiving LCT (*n* = 43) had longer progression-free survival (HR = 0.58, 95% CI 0.40–0.85, *p* = 0.01) and overall survival (HR = 0.49, 95% CI 0.30–0.79, *p* < 0.0001).

**Conclusion:**

ORD occurs with a clinically relevant frequency among PD-1/PD-L1 inhibitor-treated metastatic NSCLC patients and LCT may provide extra survival benefits in those with ORD.

**Supplementary Information:**

The online version contains supplementary material available at 10.1007/s00262-024-03720-7.

## Introduction

The advent of immunotherapy has revolutionized the management of metastatic non-small cell lung cancer (NSCLC). PD-1/PD-L1 inhibitor monotherapy or its combination with chemotherapy is now established as the first-line standard of care for metastatic NSCLC patients lacking driver mutations [1–3]. However, only a subset of patients benefit from immunotherapy [4], and many initially responsive patients eventually develop acquired resistance due to complex mechanisms [5]. This situation complicates the achievement of a universally effective salvage systemic therapy, prompting the exploration of combination strategies to mitigate and counteract resistance in patients undergoing immunotherapy [6].

The concept of oligo-residual disease (ORD), inspired by the oligo-metastasis paradigm initially described in 1995 [7], has garnered increasing attention in recent years [8–10]. Local consolidative therapy (LCT), particularly radiotherapy, has been demonstrated to enhance survival in advanced NSCLC patients with ORD following effective systemic treatment [9, 10]. At maximal response to systemic therapy, patients typically present with the lowest tumor burden, making local interventions targeting residual sites most likely to yield substantial efficacy with minimal side effects [11, 12]. Additionally, drug-resistant clones within residual tumor lesions may act as seeds for future metastases; thus, encompassing LCT may delay drug resistance onset or even extend survival [13, 14]. In the realm of targeted therapy, our prior research indicated that over one-fourth of patients with epidermal growth factor receptor (EGFR)-mutant metastatic NSCLC receiving third-generation EGFR tyrosine kinase inhibitors (TKIs) exhibited ORD at the peak response to EGFR-TKIs [10]. Patients receiving LCT in real-world settings showed notably longer survival [15], findings partly corroborated by the prospective ATOM study [16]. However, the exploration of LCT’s clinical value in immunotherapy-treated metastatic NSCLC with ORD remains unreported. Notably, radiotherapy-induced cell death can trigger immunogenic effects, potentially synergizing with immunotherapy [17–19]. Consequently, radiotherapy-based LCT might play pivotal roles, such as enhancing systemic anti-tumor immune responses and fostering long-term survival, in patients treated with PD-1/PD-L1 inhibitors for metastatic NSCLC with ORD, meriting further examination. Nonetheless, the prevalence of ORD in patients undergoing PD-1/PD-L1 inhibitor therapy and the clinical applicability of LCT in such cases remain largely unexplored.

This multicenter retrospective study extensively analyzes tumor regression patterns at the optimal response to PD-1/PD-L1 inhibitors and instances of treatment failure in patients with ORD. Additionally, it investigates the clinical significance of LCT in patients exhibiting ORD.

## Materials and methods

### Patient

Patients with metastatic NSCLC treated with PD-1/PD-L1 inhibitors, either as monotherapy or in combination with other drugs, between May 2018 and December 2021 at three institutions (Fudan University Shanghai Cancer Center, Fudan University Zhongshan Hospital, and Tongji Hospital affiliated with Tongji Medical College of Huazhong University of Science and Technology) were retrospectively reviewed, as previously described [20]. Eligible patients included those with at least one measurable lesion according to the Response Evaluation Criteria in Solid Tumors guideline (version 1.1). Exclusion criteria encompassed patients who received concurrent local therapy within four weeks of initiating PD-1/PD-L1 therapy. Neoplasm staging adhered to the eighth edition of the American Joint Committee on Cancer staging manual. The study received ethical approval from the institutional review boards of all three medical centers (Approval number: 2012228-3).

LCT, encompassing surgery, radiotherapy, and radiofrequency ablation, targeted residual tumor lesions post-effective PD-1/PD-L1 inhibitor treatment and prior to initial disease progression in a subset of patients (the LCT group). In contrast, the non-LCT group consisted of patients receiving only anti-PD-1/PD-L1 therapy without any loco-regional intervention before disease progression. For patients with stable ORD, systemic therapy continued until disease progression or the emergence of unacceptable toxicities. The decision to administer LCT was at the discretion of the treating physicians, and pertinent data were retrospectively collected for this study.

### Follow-up and response assessment

Baseline imaging for all patients prior to initiating immunotherapy included enhanced computed tomography (CT) of the chest, enhanced magnetic resonance imaging (MRI) or CT of the head, enhanced CT or ultrasound of the abdomen, and a whole-body bone scan, or whole-body positron emission tomography (PET)/CT plus enhanced MRI or CT of the head. The patients were typically followed up every 2–3 months in accordance with the institution’s standard of care and relevant guidelines. During each follow-up, comprehensive radiographic assessments were conducted, encompassing chest CT scans, CT scans or ultrasonography of the abdominal and cervical regions. Meanwhile, for those with baseline brain metastasis, brain MRI or CT was performed at each radiographic follow-up. For those with baseline bone metastasis, whole-body bone scan was generally performed every 6 months. Nevertheless, PET/CT was not mandatory and performed at the treating physicians’ discretion. All serial imaging for each patient was meticulously reviewed by a senior radiologist. Telephone follow-ups were conducted as needed. The data cut-off date was 31 December 2023.

### Definition of ORD and pattern of failure analyzes

The imaging scans of patients who achieved a partial response (PR) or stable disease (SD) as their best response to anti-PD-1/PD-L1 therapy were meticulously analyzed. Those with residual tumor distribution confined to three organs and a maximum of five lesions amenable to radical local treatment were categorized as having ORD [10, 15, 21]. Baseline oligometastatic disease (OMD) was characterized by ≤ 5 metastases and involvement of ≤ 3 organs, where all metastatic sites, including primary tumors and lymph nodes, were amenable to radical local treatment [7, 21]; cases not meeting these criteria were classified as baseline multiple metastatic disease (MMD).

Patterns of initial progressive disease (PD) were stratified into three types based on the relationship between progressive and residual tumor lesions. Residual lesion progression (RP) was identified when disease progression occurred exclusively at residual lesions. New lesion progression (NP) referred to the emergence of new tumors that were not present at the time of the best response to anti-PD-1/PD-L1 therapy. Cases where initial disease progression involved both residual and new lesions were defined as both lesion progression (BP).

### Statistical analyzes

Baseline characteristics of the study were summarized using descriptive statistics and compared via chi-square contingency analyzes. Progression-free survival (PFS) was defined as the duration from the commencement of immunotherapy to the occurrence of disease progression or death due to any cause. Overall survival (OS) was calculated from the start of immunotherapy to death from any cause. The Kaplan–Meier method, accompanied by the log-rank test, was employed to assess PFS and OS. To evaluate the impact of LCT on PFS and OS in patients with ORD, Cox proportional hazard models were used within various subgroups. Adverse events (AEs) were graded using the National Cancer Institute Common Terminology Criteria for Adverse Events (CTCAE) version 5.0.A *p* value of < 0.05 was considered statistically significant. All analyzes were performed using the Statistical Package for the Social Sciences (SPSS) software (version 20.0, SPSS Inc., Chicago, IL, USA) and GraphPad Prism (version 7.00) for Windows (GraphPad Software).

## Results

### Patient characteristics

**Fig. 1:**
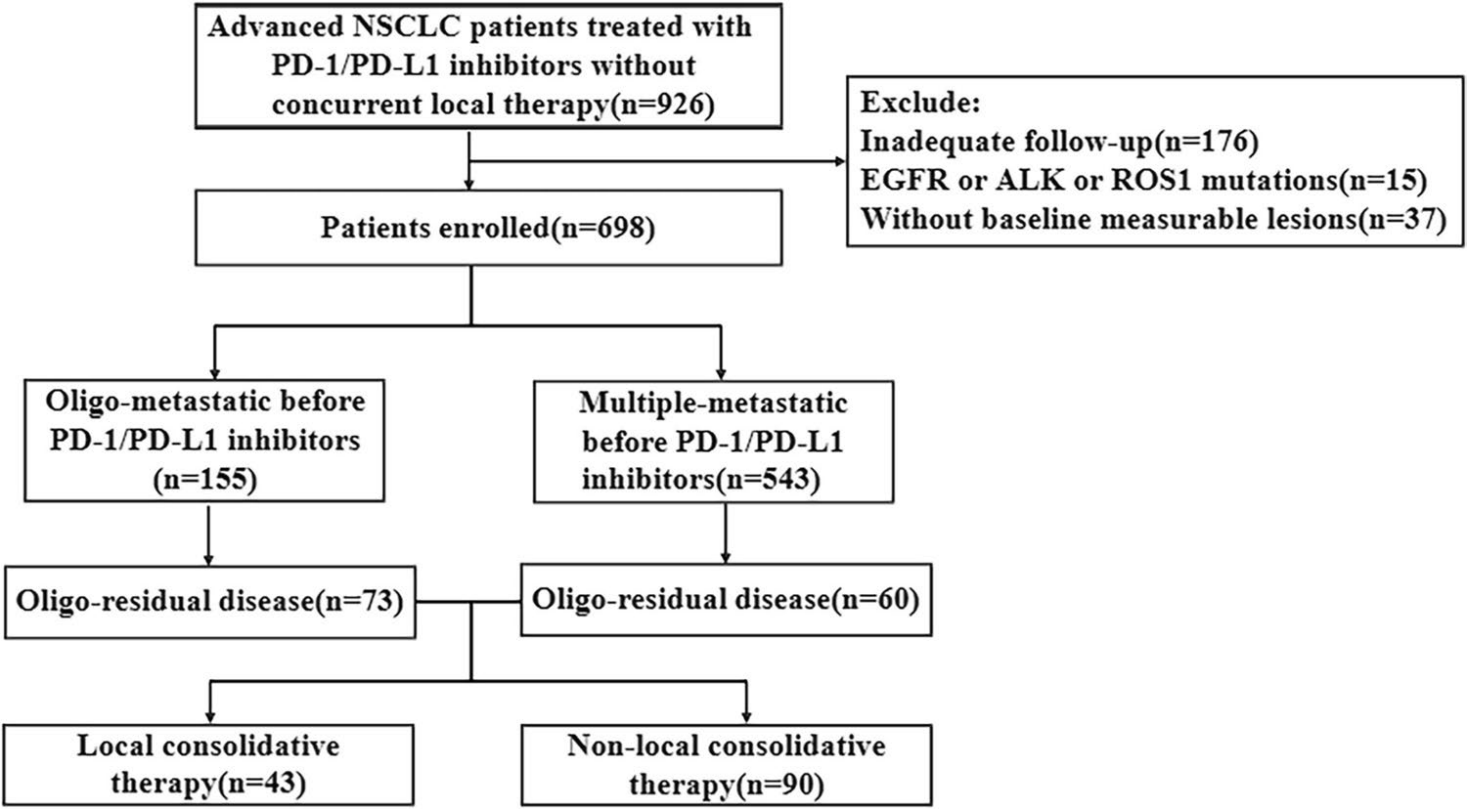
Flowchart of patient enrollment. NSCLC: non-small cell lung cancer, PD-1/PD-L1: programmed death receptor 1/programmed death receptor ligand 1. EGFR: epidermal growth factor receptor, ALK: anaplastic lymphoma kinase, PD: progressive disease, PR: partial response, SD: stable disease

Between May 2018 and December 2021, a total of 926 patients with metastatic NSCLC undergoing PD-1/PD-L1 inhibitor treatment across three academic centers were retrospectively reviewed. Out of these, 698 patients were ultimately included in the study (as shown in Fig. 1). Among the included patients, OMD and MMD were identified in 155 (22.2%) and 543 (77.8%) patients, respectively. Comprehensive demographic data and baseline disease characteristics for these 698 patients are detailed in Table 1.

**Table 1:** Patient’s baseline characteristics (*n* = 698)

	*n* (%)
Age, y	
≤ 60	299 (42.8)
> 60	399 (57.2)
Gender	
Male	565 (80.9)
Female	133 (19.1)
ECOG PS	
0	86 (12.3)
1	585 (83.8)
2	27 (3.9)
Histology	
SCC	253 (36.2)
AD	442 (63.3)
Others	3 (0.4)
Baseline disease pattern	
Oligo-metastasis	543 (77.8)
Multiple-metastasis	155 (22.2)
Baseline brain metastasis	
Yes	109 (15.6)
No	589 (84.4)
Baseline liver metastasis	
Yes	127 (18.2)
No	571 (81.8)
Baseline bone metastasis	
Yes	255 (36.5)
No	443 (63.5)
Treatment regimens	
Monotherapy	264 (37.8)
Combinational therapy	434 (62.2)
Treatment lines	
1st	163 (23.4)
≥ 2nd	535 (76.6)
PD-L1 status	
Negative*	58 (8.3)
Positive^#^	165 (23.6)
Unknown	475 (68.1)
Smoking history	
Yes	398 (57.0)
No	300 (43.0)

SCC, squamous cell carcinoma; AD, adenocarcinoma;others, other histologic categories; ECOG, Eastern Cooperative Oncology Group performance status; PD-L1, programmed death receptor ligand 1

^*^PD-L1 expression < 1%

^#^PD-L1 expression ≥ 1%

### Clinical features of ORD

During a median follow-up period of 31.0 months (range, 6.0–53.0 months), partial response (PR), stable disease (SD), and PD were observed as the best responses in 265 (38.0%), 258 (37.0%), and 175 (25.1%) patients, respectively. Notably, 73 (47.1%) of the 155 patients with baseline OMD and 60 (11.0%) of the 543 patients with baseline MMD developed ORD, as depicted in Fig. 1. Detailed demographics and baseline disease characteristics for the 133 ORD patients are provided in Supplementary Table 1. Among them, 47 (35.3%) patients underwent monotherapy with PD-1/PD-L1 inhibitors, while the remaining 86 (64.7%) received combination therapy. Additionally, PD-1/PD-L1 inhibitors were utilized as first-line therapy in 52 (39.1%) patients and as second-line or subsequent therapy in 81 (60.9%) patients, as shown in Supplementary Fig. 1. Cox regression analyzes were conducted to identify predictors of ORD. Univariate analysis indicated significant associations between treatment lines (first-line vs. second or subsequent line) and baseline disease states (OMD vs. MMD) with the development of ORD (*p* ≤ 0.001, Table 2). Multivariate analysis further identified first-line immunotherapy and baseline OMD as independent predictors of ORD (Table 2).

**Table 2:** Logistic regression analyzes for predictors of ORD

	Univariate analysis			Multivariate analysis		
	OR	95%CI	*p*	OR	95%CI	*p*
Sex (female *vs.* male)	1.103	0.688–1.769	0.684			
Age (≤ 60 *vs.* > 60)	1.258	0.854–1.852	0.245			
ECOG (0 *vs.*1 ~ 2)	0.956	0.592–1.542	0.853			
Histology (SCC *vs.* AD *vs.* others)	0.075	0.480–1.036	0.073			
Smoking (never *vs.* ever)	1.006	0.687–1.474	0.975			
Oligo-metastatic (Yes *vs.* No)	0.14	0.092–0.211	< 0.001	0.119	0.076–0.185	< 0.001
Baseline brain metastasis (Yes *vs*. No)	1.407	0.409–4.839	0.588			
Baseline liver metastasis (Yes *vs*. No)	2.104	0.668–6.629	0.204			
Baseline bone metastasis (Yes *vs*. No)	0.88	0.344–2.250	0.789			
PD-L1 status (-* *vs*. + ^#^ *vs*. unknown)	0.781	0.590–1.034	0.085			
Treatment lines (1st *vs.* ≥ 2nd)	0.321	0.215–0.481	< 0.001	0.251	0.159–0.397	< 0.001
Immune monotherapy (Yes *vs.* No)	1.141	0.770–1.692	0.512			

SCC, squamous cell carcinoma; AD, adenocarcinoma;others, other histologic categories; ECOG, Eastern Cooperative Oncology Group performance status; PD-L1, programmed death receptor ligand 1

^*^PD-L1 expression < 1%

^#^ PD-L1 expression  ≥ 1%;ORD, oligo-residual disease

Among the ORD patients, the median time to maximal response was 4.0 months (range 1.0–27.0 months). Regarding residual organ involvement, 50 (37.6%) patients had involvement in a single organ, while 83 (62.4%) had 2–3 involved organs, as illustrated in Supplementary Fig. 2A. In terms of residual tumor lesions, 36 (27.1%) patients had a single lesion, and 97 (72.9%) had 2–5 lesions, as demonstrated in Supplementary Fig. 2B. The lungs were the most common site of residual disease, detailed in Supplementary Fig. 2C and D.

### Pattern of treatment failure among patients with ORD

Of the 133 patients diagnosed with oligo-residual disease (ORD), 43 (32.3%) underwent one or more forms of LCT, which included radiotherapy (39 patients), hepatic radiofrequency (3 patients), and surgery (3 patients), as detailed in Table 3. Notably, 24 of these patients received either stereotactic or hypo-fractionated radiotherapy. The baseline characteristics were largely comparable between the LCT and non-LCT groups, except for a higher prevalence of smoking history in the LCT group (58.1% vs. 35.6%, as shown in Supplementary Table 1).

**Table 3:** Detailed information of local consolidative therapy in those with ORD

	All (*n* = 43) *n*%*
Surgical resection	3 (7.0)
Thoracic surgery	1 (2.3)
Neurosurgery	2 (4.7)
Hepatic radiofrequency ablation	3 (7.0)
Radiation therapy^#^	39 (90.7)
Radiotherapy type	
Conventional radiotherapy	19 (44.2)
Hypofractionated radiotherapy	14 (32.6)
Stereotactic radiotherapy	10 (23.3)
Radiotherapy organs	
Thoracic radiotherapy	22 (51.2)
Bone radiotherapy	8 (18.6)
Cranial radiotherapy	6 (14.0)
Irradiation to other sites	7 (16.3)
Dose fractions	
60 Gy/30Fx	8 (18.6)
24 Gy/3Fx	8 (18.6)
30 Gy/10Fx	8 (18.6)
50 Gy/25Fx	7 (16.3)
Others	8 (18.6)
Biologically effective doses	
≥ 50 Gy	20 (46.5)
< 50 Gy	19 (44.2)
Radiotherapy intention	
Palliative	21 (48.8)
Ablative	18 (41.8)

^*^Certain patients may have received two or more local consolidation treatments. ORD, oligo-residual disease

^#^Certain patients received more than one types of radiotherapy targeting different tumor sites with distinct doses

**Fig. 2:**
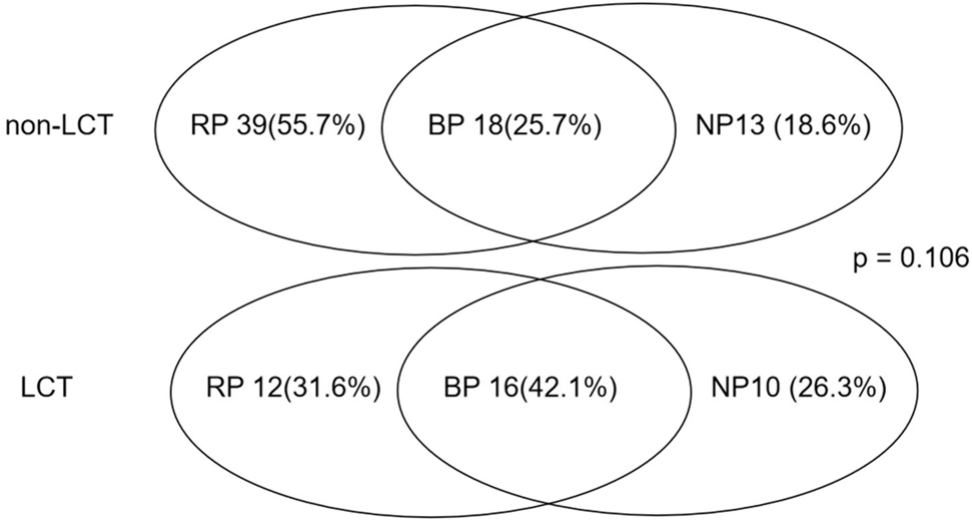
Patterns of disease progression in patients with oligo-residual disease. Venn diagram of patterns of disease progression in LCT group (upper) and non-LCT group (lower) of patients with ORD at maximal response. LCT: local consolidation treatments, ORD: oligo-residual disease, RP: residual lesion progression, NP: new lesion progression, BP: both lesion progression

By the time of data cut-off, 108 (81.2%) of the 133 ORD patients experienced initial PD. In fact, 74 (68.5%) of the 90 patients in the non-LCT group and 34 (31.5%) of the 43 patients in the LCT group developed their initial PD. Specifically, RP occurred in 51 (47.0%) patients, NP in 23 (21.3%), and BP in 34 (31.5%). In comparison, 56 (62.2%) of the 90 patients in the non-LCT group developed initial PD. The distribution of RP, NP, and BP between the LCT and non-LCT groups was not significantly different (*p* = 0.122), as depicted in Fig. 2.

### Clinical outcomes among patients with ORD

**Fig. 3:**
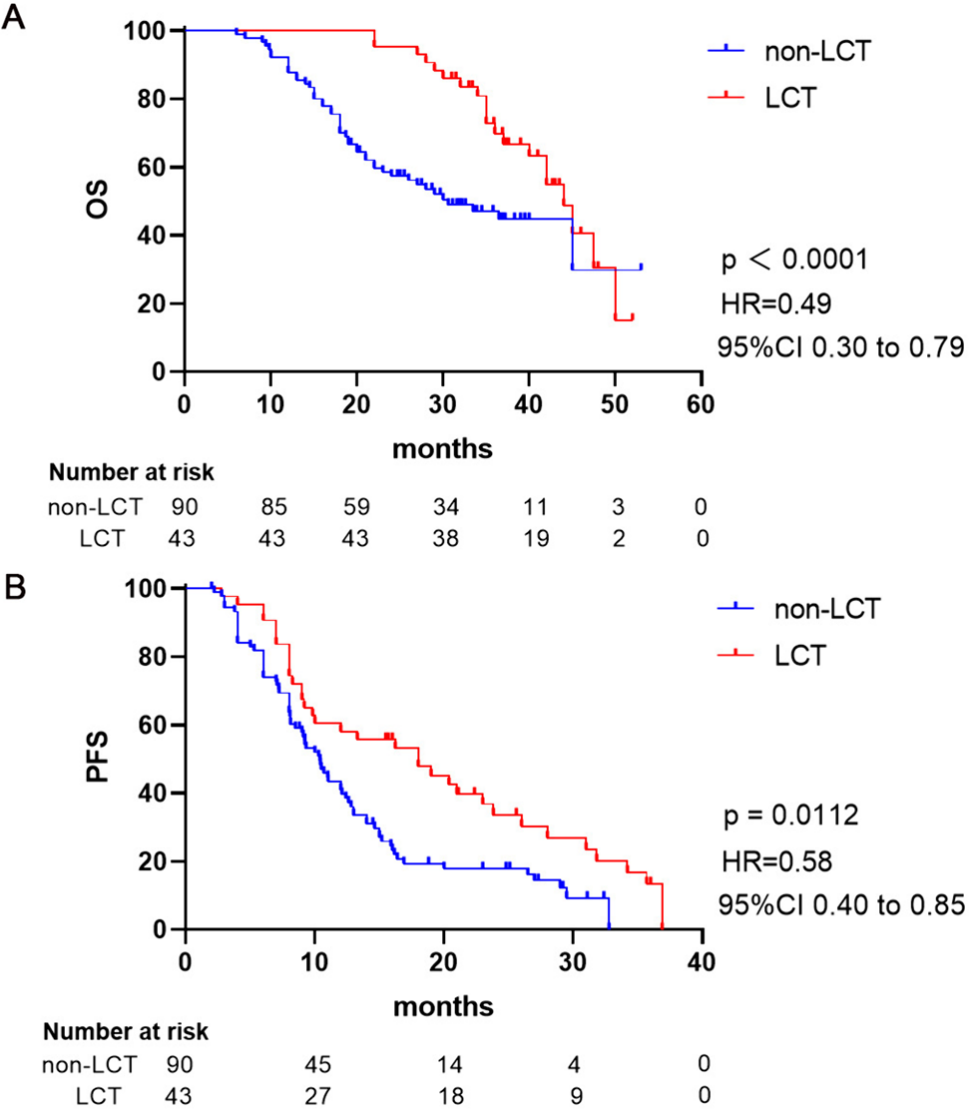
Kaplan–Meier estimates of PFS and OS. Kaplan–Meier estimates of PFS (A) and OS (B) for patients with ORD stratified by LCT status (LCT vs. non-LCT). LCT: local consolidation treatments, PFS: progression-free survival, OS: overall survival, BMs: brain metastases, LCT: local consolidative therapy

Among the 133 patients with ORD, the median PFS was 12.0 (95% CI 9.09–12.99) months. Of them, the median PFS was 18.0 (95% CI 9.87–26.13) and 10.0 (95% CI 8.73–12.07) months among those with and without LCT, respectively. Patients who underwent LCT experienced a significantly longer PFS (*p* = 0.01, Hazard Ratio [HR] = 0.58, 95% CI: 0.40–0.85), when compared to those who did not receive LCT (Fig. 3A).

By the time of data cut-off, 67 (50.4%) patients had died and the median OS was 40.0 (95% CI 34.61–45.39) months in the entire cohort of patients with ORD. Of them, the median OS was 44.0 (95% CI 39.78–48.22) and 30.0 (95% CI 20.67–40.53) months among those with and without LCT, respectively. Notably, LCT was associated with a significant extension in OS (HR = 0.49, 95% CI 0.30–0.79, *p* < 0.0001), depicted in Fig. 3B. Furthermore, LCT was identified as an independent predictor of improved PFS and OS following Cox regression analyzes, as detailed in Supplementary Tables 2 and 3.

Meanwhile, grade 3–5 treatment-related AEs among the 133 patients with ORD were summarized in supplemental Table 4. The incidence of grade 3–5 treatment-related AEs were similar among those with or without LCT.

## Discussion

Local consolidative therapy is gaining traction among metastatic non-small cell lung cancer (NSCLC) patients with ORD following effective systemic therapy. Previous studies, including our own, have provided critical evidence supporting the survival benefits of LCT in the context of chemotherapy and targeted therapy [1–3, 10, 15]. Given the potential synergistic effects between radiotherapy and immunotherapy, the role of consolidative radiotherapy in immunotherapy-treated patients with ORD demands thorough investigation. To our knowledge, this study is the first to examine the clinical characteristics of ORD and assess the clinical value of LCT in metastatic NSCLC patients treated with PD-1/PD-L1 inhibitors, offering preliminary evidence for the potential clinical significance of consolidative radiotherapy in this patient group.

The clinical occurrence of ORD is notable in metastatic NSCLC patients treated with EGFR tyrosine kinase inhibitors (EGFR–TKIs). Taichi Miyawaki’s analysis of 207 patients with advanced EGFR-mutated NSCLC treated with first-line EGFR–TKIs over seven years revealed that 32% experienced ORD [22]. Similarly, our previous study found that 26.8% of patients with metastatic NSCLC treated with Osimertinib developed ORD at maximal response, suggesting their suitability for consolidative stereotactic radiotherapy [10]. However, this study marks the first instance of determining ORD frequency (19.1%) in the era of immunotherapy using real-world data from a sizeable sample, a finding that requires validation in prospective trials.

In targeted therapies for NSCLC, about half of the patients experience initial progressive disease (PD) exclusively from residual tumor lesions [10], a finding mirrored in our study among patients receiving immunotherapy. Notably, substantial intra-tumor and inter-tumor heterogeneities, particularly in genetic aberrations and immune microenvironments, are prevalent in metastatic NSCLC [23–26]. These heterogeneities can result in varying drug sensitivities across tumor lesions, with drug-resistant sub clones potentially becoming the source of residual disease and, ultimately, the seed for PD. Accumulating evidence indicates that most patients who acquire resistance to PD-1/PD-L1 inhibitors develop oligo-progressive disease originating from pre-existing tumor sites [27]. Theoretically, preemptive local therapy aimed at eradicating these residual lesions could prevent the systemic spread of resistant clones. Additionally, local consolidative therapy (LCT) plays a critical role in preventing local symptoms and complications associated with tumor growth. Therefore, local interventions targeting residual lesions prior to disease progression are potentially valuable in managing metastatic NSCLC after effective systemic treatment.

LCT, particularly beneficial in patients under conventional therapy [9, 15, 28, 29], is now, for the first time in this study, also shown to offer additional survival benefits for metastatic NSCLC with ORD in the context of immunotherapy. As a primary local treatment, radiotherapy, when combined with immunotherapy, can significantly enhance the antitumor immune response, leading to improved tumor control [30]. For instance, radiotherapy not only increases tumor cell susceptibility to T-cell-mediated attacks but also induces immunogenic cell death. This process involves promoting the release of tumor antigens from dead cells, enhancing MHC class I expression, and upregulating immune regulatory cell surface molecules [31]. Furthermore, radiotherapy upregulates PD-L1 expression to enhance radio-sensitization and counteract adaptive immune resistance [32, 33]. The integration of radiotherapy with immunotherapy in NSCLC patients has thus garnered extensive interest [34]. Additionally, in immunotherapy, a lower tumor burden correlates with higher treatment efficacy in lung cancer and other solid tumors [35, 36]. Consequently, effective debunking by LCT may help eliminate drug-resistant tumor clones and bolster systemic antitumor immune responses in patients with ORD. In our study, LCT was demonstrated to extend both progression-free survival (PFS) and overall survival (OS) using real-world data, underscoring the need for further validation in future research.

Compared to salvage local therapy in patients with oligo-progressive disease [37–40], local consolidative therapy (LCT) in those with oligo-residual disease (ORD) may offer several advantages. Our recent study in patients with EGFR-mutant advanced NSCLC receiving Osimertinib showed that, compared to salvage radiotherapy in patients with oligo-progressive disease, consolidative radiotherapy targeting EGFR-TKI resistant clones at all oligo-residual tumor sites potentially offered a survival advantage [10]. Similarly, in the context of immunotherapy, consolidative local therapy may have benefits over salvage local therapy. Firstly, tumor burden is at its lowest following maximal response to PD-1/PD-L1 inhibitors, making consolidative local therapy at this juncture potentially more effective and less toxic [11, 12]. Secondly, while the majority of immunotherapy patients develop oligo-progressive disease, a subset may experience multiple progressive diseases with rapid deterioration, rendering them unsuitable for salvage local therapy and leading to poorer prognosis. In our study, preliminary survival benefits were observed in patients receiving LCT compared to those who did not, among whom salvage local therapies could have been administered. Future clinical trials are necessary to compare the safety and efficacy of LCT and salvage local therapy in metastatic NSCLC patients receiving immunotherapy.

Our study has several limitations. Firstly, due to the nature of retrospective design and a relatively small number of patients with ORD in the current study, the potential clinical value and survival benefit of LCT among patients with ORD should be interpreted with caution since considerable selection bias could be existed. Although the baseline disease characteristics between the LCT group and the non-LCT group were generally balanced, there may be some crucial baseline parameters missed. Nevertheless, relevant clinical data about the clinical value of LCT in immunotherapy-treated NSCLC is currently scarce. There has been a growing need to obtain a proper combinational strategy to overcome acquired resistance in NSCLC patients receiving PD-1/PD-L1 inhibitors. This study provided preliminary but valuable information about the rationale and potential clinical value of LCT in PD-1/PD-L1-inhibitor-treated NSCLC with ORD. Secondly, it was difficult for us to collect the comprehensive and accurate information about the safety profiles of the combinational therapy of LCT and immunotherapy. In the current study, only grade 3–5 treatment-related AEs were documented, which were generally consistent with previous studies reporting promising results about the safety profiles of the combination therapy of PD-1/PD-L1 inhibitors with radiotherapy, especially hypo fractionated radiotherapy and SBRT [41–43]. In a recent phase 2 trial, upfront local ablative therapy consisting of radiotherapy, surgery and radiofrequency ablation, combing with pembrolizumab, resulted in generally comparable incidence of treatment-related toxicities with those receiving pembrolizumab monotherapy in oligo-metastatic NSCLC [41]. Grade ≥ 2 treatment-related adverse events occurred in 25% of the patients receiving hypofractionated radiotherapy and immunotherapy in a pooled analysis of two prospective trials, which was generally manageable [44]. Similarly, a meta-analysis including 51 studies with 15,398 patients found comparable grade 3–4 toxicities in those receiving both immunotherapy and radiotherapy (16.3%; 95% CI, 11.1–22.3%) and those receiving immunotherapy alone (22.3%; 95% CI, 18.1–26.9%) [43].

## Conclusions

Oligo-residual disease (ORD) was observed in approximately 20% of patients undergoing treatment with PD-1/PD-L1 inhibitors. Moreover, local consolidative therapy (LCT) appears to offer additional survival benefits for those exhibiting ORD following effective PD-1/PD-L1 inhibitor therapy. Nonetheless, the validation of this observation in future prospective studies with larger sample sizes is imperative.

### Supplementary Information

Below is the link to the electronic supplementary material.

Supplementary file1 (TIF 7500 KB)

Supplementary file2 (TIF 9153 KB)

Supplementary file3 (XLSX 15 KB)

Supplementary file4 (XLSX 13 KB)

Supplementary file5 (XLSX 13 KB)

Supplementary file6 (XLSX 10 KB)

## Data Availability

The data that support the findings of this study are available from the corresponding author, upon reasonable request.
