# Supplementary figures and images for: Oligo-residual disease in PD-1/PD-L1 inhibitor-treated metastatic non-small cell lung cancer: incidence, pattern of failure, and clinical value of local consolidative therapy
Source: Cancer Immunol Immunother. 2024 Jun 4;73(8):140. doi: 10.1007/s00262-024-03720-7 (PMC11150343; doi:10.1007/s00262-024-03720-7)

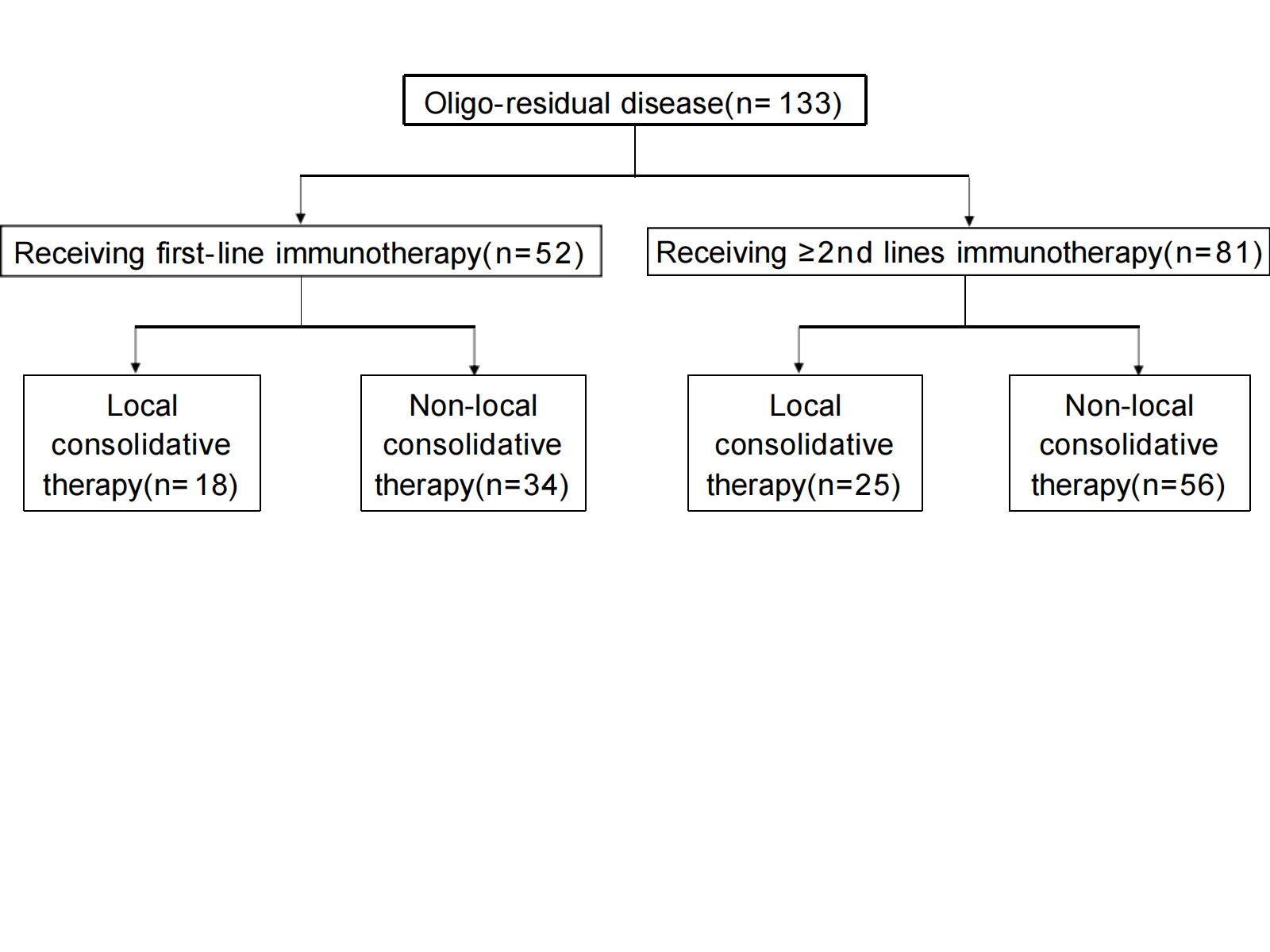

Supplement: Supplementary file 1 — Supplementary file1 (TIF 7500 KB) [file 262_2024_3720_MOESM1_ESM.tif]

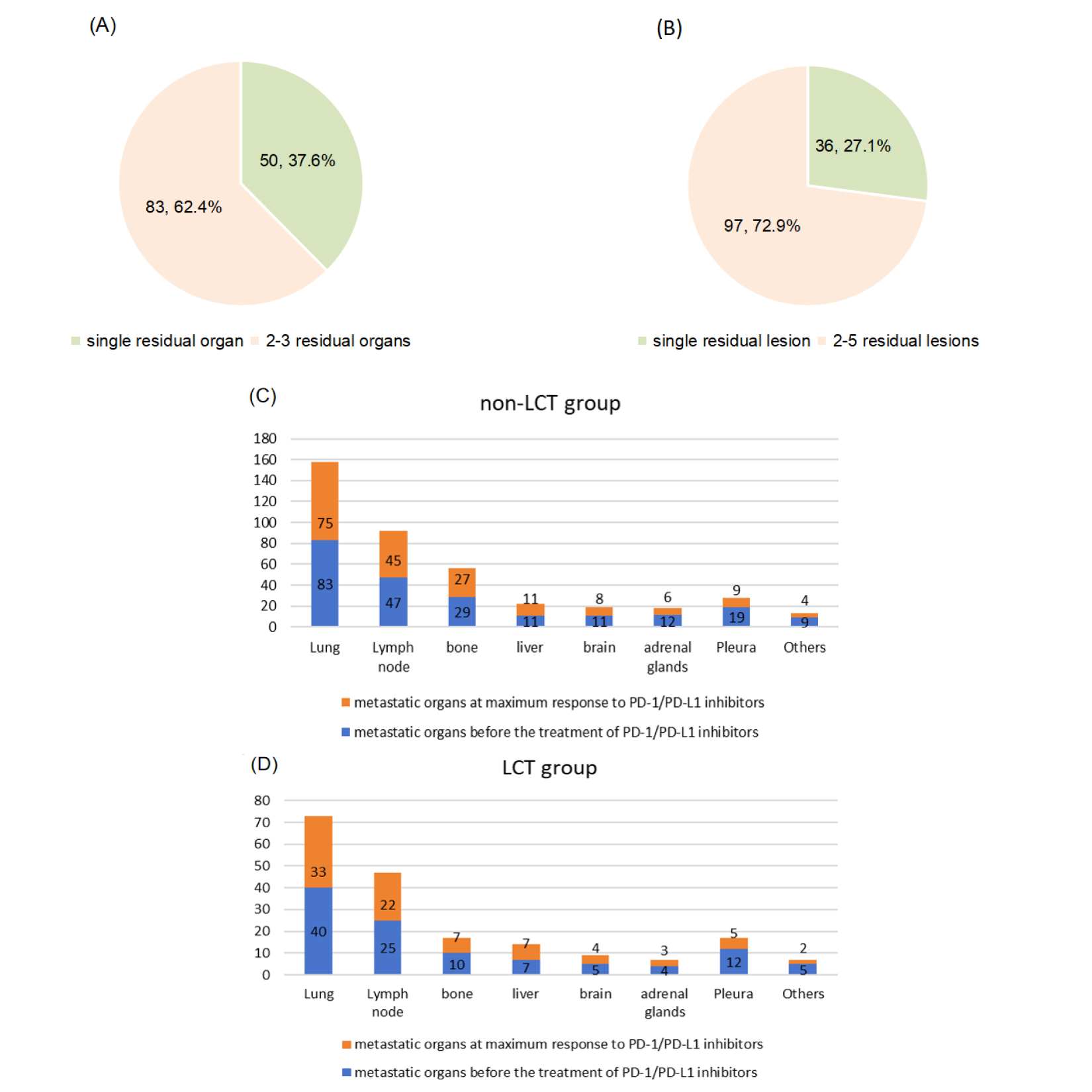

Supplement: Supplementary file 2 — Supplementary file2 (TIF 9153 KB) [file 262_2024_3720_MOESM2_ESM.tif]
